# Acute Intestinal Obstruction with Abnormal Features

**Published:** 1908-01-25

**Authors:** 


### Points in Surgery.

### ACUTE INTESTINAL OBSTRUCTION WITH ABNORMAL FEATURES.

The diagnosis in cases of acute abdominal disease presents one of the most perplexing problems in surgery. The case about to be described illustrates one aspect of this problem—the difficulty of diagnosing between acute abdominal disease of inflammatory origin and that which is due to a mechanical cause. A further complication exists in the fact that an inflammatory condition may be secondary to a mechanical obstruction, as, for instance, when perforation of the intestine and peritonitis are consecutive to strangulation of the bowel in a retro-peritoneal hernia.

The patient, a man aged forty-one, had always enjoyed excellent health, except for what he described as an attack of "Colonial fever" in Australia many years previously. Since then he had followed a laborious occupation without a day's illness. One morning at 4 a.m. he was seized with sudden violent abdominal pain of an excruciating nature, which was more or less constant, but underwent exacerbations which literally doubled him up. He had not been constipated before the attack. He vomited once or twice in the first few hours after its onset. His temperature was normal, and his pulse of good quality (100). It appeared probable that he was suffering from an acute attack of colic. His face was contorted, and his abdomen was kept rigid on account of the pain, and he declared that it was tender on palpation. It was not distended. He referred the pain at one time to the epigastrium, and at others to the right or left iliac fossa indiscriminately, so that the history obtained from him was of little value. He was not, and declared he never had been, jaundiced; nor had he ever passed any blood in his water. He had no sign of inguinal or femoral hernia. He was given one-sixth of a grain of morphia without relief of his symptoms, and an enema with little or no result. Later he was given twenty minims of tincture of opium: this was vomited. During the course of the day he had two more enemata, each without result. He passed no water, so that it could not be determined whether he had hæmaturia at the moment or not. At 11 p.m., as his symptoms continued, chloroform was administered, in the hope that when he came round his pain might have abated. The opportunity was taken of passing a catheter and withdrawing his urine. It was found to contain no blood, but some reducing body was present. Examination of the abdomen under the anæsthetic gave no further information. It was still rather rigid, but it was resonant all over, and no mass was felt. Just before the chloroform was given, his pulse, which was still good in quality, was 132; under the anæsthetic it dropped to 104. When he came round he vomited again several times clear green fluid which appeared to be gastric or biliary. The pain soon returned, and at midnight he was given another quarter of a grain of morphia *sub cutem*, and under the influence of this he slept till the morning. When the effect of the morphia passed off, his pain returned, and his general condition appeared to be worse. His face began to have an abdominal aspect, his temperature was 100, and his pulse, which was now not so strong, was 140. His abdomen was somewhat distended and inclined to be tympanitic on percussion—at any rate, the note was distinctly higher than it had been the night before. In view of this evidence it was considered that the patient probably had some peritonitis, and an operation was advocated. The abdomen was opened about thirty-six hours after the onset of the atack, and some free serous, but not purulent, fluid found in the abdomen. The ileum was constricted by an omental band not very far from its termination in the cæcum, the distal end of the omental tag being adherent to the parietal peritoneum of the anterior abdominal wall. The intestine above was distended and slightly injected; that below was pale and collapsed. There was no gangrene and no perforation at the point where it had been bound down; nor was there any peritonitis, although the serous coat of the bowel felt sticky, and it is likely that peritonitis would have supervened if operation had been delayed. Immediately the constriction was released the contents passed along into the collapsed gut.

It will be seen that in this case the diagnosis was more than ordinarily obscure. The violent nature of the pain, and its sudden onset, led to the belief that the man's condition was due to the passage of a calculus, in spite of the absence of both hæmaturia and jaundice. This is found to be the cause in most cases of excruciating abdominal pain which doubles the patient up. Patients with peritonitis complain of pain, it is true, but they do not move their body: they roll their head from side to side, and look acutely ill. This man flung himself about, and at times assumed the genupectoral position, which, he said, afforded him some ease. Intestinal obstruction was considered. In favour of this diagnosis was the failure of the enemata to produce any result and the vomiting. But the vomit was never fæcal, and the abdomen was not distended; nor did he at any time present the symptoms of shock associated with sudden strangulation of the bowel. Before the operation was performed, it was thought probable that peritonitis had set in. This presumption was founded on the tympanites of the abdomen, the weak, rapid pulse, and the rise of temperature. Whereas the true solution of the mystery was that the patient had acute obstruction to the passage of intestinal contents from the outset, without definite strangulation, and that his paroxysms of pain were due to the ineffectual efforts of the small intestine to drive on its contents. The rapid pulse was probably a reflex phenomenon due to the pain. Whether he would eventually have developed peritonitis from gangrene of the gut, and if so, how soon, are questions that it is almost impossible to answer.

